# Brain-Derived Neurotrophic Factor, Depression, and Physical Activity: Making the Neuroplastic Connection

**DOI:** 10.1155/2017/7260130

**Published:** 2017-08-08

**Authors:** Cristy Phillips

**Affiliations:** Department of Physical Therapy, A-State, Jonesboro, AR, USA

## Abstract

Brain-derived neurotrophic factor (BDNF) is a neurotrophin that is vital to the survival, growth, and maintenance of neurons in key brain circuits involved in emotional and cognitive function. Convergent evidence indicates that neuroplastic mechanisms involving BDNF are deleteriously altered in major depressive disorder (MDD) and animal models of stress. Herein, clinical and preclinical evidence provided that stress-induced depressive pathology contributes to altered BDNF level and function in persons with MDD and, thereby, disruptions in neuroplasticity at the regional and circuit level. Conversely, effective therapeutics that mitigate depressive-related symptoms (e.g., antidepressants and physical activity) optimize BDNF in key brain regions, promote neuronal health and recovery of function in MDD-related circuits, and enhance pharmacotherapeutic response. A greater knowledge of the interrelationship between BDNF, depression, therapeutic mechanisms of action, and neuroplasticity is important as it necessarily precedes the derivation and deployment of more efficacious treatments.

## 1. Introduction

Major depressive disorder (MDD) is a leading cause of global disease burden that affects over 300 million persons worldwide [1–3]. Pathognomonic features of this complex mental illness include the persistence of one or more episodes of sadness or anhedonia in a two-week period, along with the manifestation of cognitive and somatic symptoms (e.g., changes in appetite, sleep patterns, energy level, concentration, or physical activity; feelings of worthlessness and guilt; and suicidal thoughts or behaviors) [4]. The disorder can consist of a single episode or several recurrent episodes. Direct and indirect costs of treating MDD in the United States exceed $210 billion annually [5]. The high costs to individuals and society demand efficacious treatments, yet 30% of patients fail to respond to current pharmacotherapeutics and 70% do not achieve complete remission [6]. Disenchantment with the ability of extant drugs to mitigate symptoms in a significant proportion of persons, the undesirable side effects, and the high economic and social cost to society have prompted a diversification of the search for effective treatment options. Of the alternative therapeutics garnering attention, physical activity (PA) has shown clear and consistent promise for mitigating depressive neurobiology through mechanisms that involve brain-derived neurotrophin factor (BDNF).

Because it is important that clinicians and scientists understand the means by which PA can be used to optimize BDNF levels and mitigate pathophysiological substrates of depression, from both a self- and patient-education perspective, the aims of this review are to (1) explicate the putative neurobiological mechanisms involved in MDD and how trophic factors relate to those mechanisms, (2) review clinical and preclinical evidence of altered BDNF in persons with MDD, (3) discuss the relationship of BDNF to neuroplasticity, (4) discuss the effect of PA on BDNF, and (5) highlight current and future implications for clinicians and scientists.

## 2. Neurobiology of Depression

Neuroimaging studies of depression and surgical lesion studies that induce or mitigate depressive symptoms have been used to elucidate mood circuits [7, 8]. Comprising these circuits are several brain structures and regions, particularly the dorsal prefrontal cortex, ventral prefrontal cortex, anterior cingulate gyrus, amygdala, hippocampus, striatum, and thalamus [9–11]. Several pathophysiological processes are implicated in mood circuit and structure dysfunction [7, 12], including those related to genetic, epigenetic, and environmental factors. Results from a twin study suggest that the heritability of MDD is approximately 40% [13]. Preclinical studies have implicated epigenetic mechanisms by demonstrating that maternal behavior alters the function of stress-related genes [14] just as the administration of antidepressant drugs alters DNA regulation [15]. Other studies have shown that the depletion of neurotransmitters (e.g., dopamine, serotonin, and norepinephrine) contributes to depressive symptoms [16, 17] by altering glutamate and *γ*-aminobutyric acid (GABA) signaling. Accordingly, therapeutic agents were derived to either inhibit neuronal reuptake or inhibit degradation of monoamines in the synaptic cleft, actions aimed at increasing monoamine transmission. Yet monoamine depletion failed to produce depressive symptoms in persons who were healthy or worsen the severity of symptoms in persons with MDD [18]. Moreover, recent preclinical and clinical investigation demonstrated that ketamine, an NMDA receptor antagonist, induced rapid antidepressant effects through different mechanisms than monoamine reuptake inhibitors [18]. Subsequent study of signaling mechanisms that underlie the rapid antidepressant effects of ketamine has implicated BDNF and its ability to induce neuronal network alterations [18, 19].

**Figure 1:**
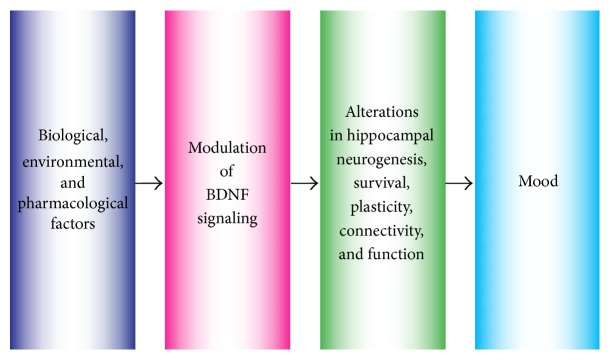
Endogenous and exogenous factors modulate BDNF levels to effectuate changes in the hippocampus and mood. Environmental stress—along with biological, genetic, and pharmacological factors—modulates BDNF levels and synaptic plasticity in various regions of the brain, including the hippocampus. Decrements in BDNF levels can confer vulnerability for hippocampal dysfunction and loss of emotional regulation. Conversely, antidepressant administration and voluntary PA optimize BDNF levels in the hippocampus and mitigate mood symptoms.

The neurotrophic hypothesis of depression proposes that stress-related alterations in BDNF levels occur in key limbic structures to contribute to the pathogenic processes in MDD [19]. This notion is prefaced on evidence that neurotrophins are growth factors that play pivotal roles in the formation and plasticity of neuronal networks [20], and yet persons with MDD exhibit region-specific alterations in the level and function of BDNF. Upregulation of BDNF occurs in the amygdala and nucleus accumbens of persons with MDD whereas downregulation of BDNF occurs in the hippocampus and medial prefrontal cortex (mPFC) [21]. BDNF abnormalities also contribute to dysfunction of astrocytes and microglia in depression circuits. It has been shown that (1) persons with MDD exhibit decreased expression of glial fibrillary acidic protein and mRNA in the frontolimbic cortical region, (2) BDNF modulates glial function, and (3) antidepressant administration and deep brain stimulation mitigate glial deficits [22]. Finally, upregulation of BDNF occurs following chronic administration of antidepressants or voluntary participation in PA, consistent with the time course for the therapeutic action of antidepressants and PA [23]. Taken together, this evidence suggests that altered level and function of neurotrophins contributes to the atrophy, synaptic disconnection, and dysfunction of MD-related circuits [24, 25]. Conversely, optimization of BDNF levels facilitates synaptic plasticity and remodeling, induction of long-term potentiation (LTP), modulation of gene expression for plasticity, resilience to neuronal insults [24–26], and alleviation of depressive symptoms [27] (see Figure 1). This evidence has led to concerted efforts to better understand the actions of BDNF and how these actions can be harnessed to maintain, repair, and reorganize damaged emotional and cognitive circuits, a central goal for MDD treatment and rehabilitation.

## 3. Brain-Derived Neurotrophic Factor: Localization, Synthesis, Release, and Binding

Neurotrophins are a closely related family of proteins in the brain that contributes to the survival, growth, and maintenance of neurons [28] and participate in a variety of learning and memory functions [29]. The mammalian neurotrophins include BDNF, nerve growth factor, neurotrophin 3, and neurotrophins 4-5. Undoubtedly, the majority of the literature linking neurotrophins with depression involves the study of BDNF. BDNF has proven to be one of the most highly inducible neurotrophins with PA, prompting the focus on this neurotrophin herein.

The synthesis of BDNF occurs in both the central and peripheral nervous system by target neurons under physiologic conditions and by astrocytes following injury, inflammation, or administration of antidepressants [30–32]. In the brain, neurons are considered a significant cellular source of BDNF, and synthesis occurs in regions that participate in emotional and cognitive function (e.g., hippocampus and frontal, parietal, and entorhinal areas). Gene expression studies in humans have revealed that central BDNF is highest in the cortex, hippocampus, amygdala, basal forebrain, dorsal vagal complex, hindbrain, and midbrain [33, 34]. Several brain regions retrogradely transport BDNF from their projection areas. Raphe nuclei in the brainstem of rodents do not contain BDNF mRNA [35], but serotonergic neurons in these nuclei retrogradely transport BDNF from the frontal cortex, occipital cortex, entorhinal cortex, and amygdala (their projection areas) to their cell bodies [36, 37]. Noradrenergic neurons of the locus coeruleus retrogradely transport BDNF from the frontal and entorhinal cortex. It appears that nine different gene promoters induce the tissue-specific expression of 24 different BDNF transcripts, suggesting multilevel regulation of expression across brain regions [38]. The use of different promoters can facilitate the involvement of a myriad of transcription regulatory factors and mRNA-targeting signals, factors that shunt the translation of BDNF towards activated synaptic sites [39–41].

BDNF is synthesized as a precursor (pre-pro-BDNF protein) that results from cleavage of a 32 kDa pro-BDNF protein. Pro-BDNF can be proteolytically cleaved intracellularly by enzymes (e.g., PC7, furin, and proconvertases) and secreted as the 14 kDa mature form [42, 43] or it can be secreted as pro-BDNF and subsequently cleaved extracellularly by proteases (e.g., metalloproteinases and plasmin) [44]. Both forms of BDNF (pro-BDNF and mature) are sorted and packaged into vesicles for activity-dependent secretion [36, 45, 46]. Pro-BDNF can be internalized and stored by astrocytes and later released as the immature (pro-BDNF) or mature (BDNF) form [47].

Pro-BDNF mediates its biological actions through binding to low-affinity p75 neurotrophin receptors, whereas mature BDNF binds with higher-affinity tropomyosin-related kinase family (Trk) receptors [48]. Once bound to its cognate receptors, BDNF is internalized along with its receptor and transported via retrograde axonal transport mechanisms to the soma wherein it can initiate a multiplicity of effects within the nucleus [49]. The functional importance of differential binding to either p75 or Trk receptors is underscored by their opposing effects. Proneurotrophin binding to p75 reduces spine complexity and density [50], induces long-term depression (LTD) [51], promotes neuronal cell death [52], and facilitates the resculpting of neuronal circuits [48]. These biological actions are accomplished via activation of a receptor complex that is composed of p75 and sortilin [52, 53]. In contrast, mature neurotrophin binding to Trk receptors increases cell survival and differentiation, dendritic spine complexity, long-term potentiation (LTP) [54, 55], synaptic plasticity [56], and the resculpting of networks [57]. Localization of TrkB receptors significantly increases at synaptic sites following neuronal activity [58].

## 4. BDNF Abnormalities in Persons with Depression

There is a well-established body of clinical evidence implicating the involvement of BDNF in the pathobiology of depression [59]. Peripheral reductions in mature BDNF in serum and plasma have been noted in persons with depression [60, 61] and in cases of suicide [62, 63], and psychosocial stress appears to exert a role in these decrements [64]. Findings from a recent meta-analysis and systematic review showed significantly lower levels of serum mature and pro-BDNF in antidepressant-free patients with MDD as compared to healthy controls [65]. Notwithstanding, serum levels of BDNF tend to normalize in response to several treatments (e.g., antidepressants [66], electroconvulsive therapy [67], and PA [68]).

Central reductions in BDNF in specific brain regions have been reported also. A postmortem study of persons with MDD reported decrements in BDNF protein in the hippocampus [19], along with smaller hippocampal volumes [69]. Dunham and colleagues reported a reduction of pro-BDNF in all layers of the right hippocampus in persons with depression [70]. Postmortem hippocampal samples taken from suicide completers exhibited increased mRNA for the p75 receptor [71], intimating that LTD and pruning may underlie hippocampal pathology. Thompson and colleagues reported a reduction of BDNF mRNA in layer II of the entorhinal cortex relative to controls [72]. BDNF levels were reduced in the hippocampus of postmortem samples taken from suicide completers [71, 73]. The activity of MAP kinase signaling, a major downstream signaling pathway associated with TrkB, was reduced in persons with depression [74, 75]. Conversely, persons treated with antidepressant drugs exhibited increased BDNF expression and CREB in certain regions of the brain [76].

Further implicating BDNF with MDD are genetic [73, 77] studies demonstrating that depressive behavior is associated with altered BDNF functioning [78]. The Val66Met polymorphism in the *BDNF* gene is a common single-nucleotide variant associated with MDD [79, 80]. It has an allele frequency of 20 to 30% in Caucasian populations [81]. The Val66Met polymorphism affects intracellular packaging of the pro-BDNF polypeptide and activity-dependent release [82, 83]. This polymorphism is associated with decreased hippocampal volume in healthy persons [84–86], persons with MDD [87], and persons suffering an adverse response to stress [88]. Also, it has been shown that persons who experience early-life stress and carry the Val66Met polymorphism exhibit significantly less grey matter in the subgenual anterior cingulate cortex [89] and are at increased risk for depression [90]. The Val66Met polymorphism is also a risk factor for geriatric depression [91] and has been shown to modulate antidepressant drug efficacy in Asians [92]. Thus, this naturally occurring genetic variant of the BDNF gene may contribute to a genetic predisposition for depressive disorder.

In addition to associations of the Met66 allele with decreased hippocampal size, other studies have demonstrated reduced hippocampal activation and poorer episodic memory [83, 93]. The contribution of BDNF to mechanisms of learning and memory involves the modulation of synaptic transmission and plasticity [94, 95] and refinement of synaptic architecture [94, 96, 97] as well as activity-dependent transcription. It is generally held that activity-dependent transcription provides a mechanism by which neurons convert transient cellular changes to stable changes in brain function, particularly in memory formation. Increased calcium influx via voltage-gated Ca^2+^ channels and NMDA receptors is vital for neuronal plasticity mechanisms [98, 99]. NMDA receptor activation is particularly important for hippocampal synaptic plasticity [100] and modulation of BDNF [101], given that the latter determines the strength of existing synaptic connections and promotes the formation of new synapses. Together, this evidence suggests that alterations in BDNF function may affect activity-dependent plasticity in the hippocampus and thereby learning in memory and emotions in persons with MDD.

Knowledge of BDNF level and function is relevant for MDD research and treatment purposes for the several reasons listed.

- Altered BDNF function can contribute to an increased risk of depression and suicidal behavior [102].
- With refinement of current knowledge, BDNF may eventually serve as a biomarker of depression and suicidal behavior in persons with depression and enhance diagnostic and treatment efforts [103, 104].
- Treatment-induced normalization of BDNF may promote neural health and recovery of function from illness [105]. Additionally, the administration of BDNF-enhancing techniques (e.g., PA and transcranial stimulation) may enhance pharmacotherapeutic response [106].

## 5. BDNF in Animal Models of Depression

Stress is a well-known harbinger of depression in persons with genetic vulnerability [13, 107]. Evidence of the link between stress and depression has prompted investigators to derive animal research models of stress (e.g., immobilization, unpredictable chronic stress, foot shock, social isolation, social defeat, restraint, forced swim, and maternal deprivation) to determine a cause and effect relationship between pathology, interventions, BDNF, and depressive symptoms (for excellent reviews of depression metrics in animals, see [108–114]).

Preclinical studies have demonstrated that chronic stress and depressive-like symptoms are associated with reduced BDNF synthesis and activity of TrkB in the hippocampus and frontal cortex [19, 115, 116], making it seem plausible that decreased levels of BDNF induce a state of increased vulnerability to stress and depression. Conversely, direct infusion of BDNF into the hippocampus or midbrain yields antidepressant-like effects [117, 118]. Other studies have shown that chronic administration of antidepressants increases BDNF mRNA protein in the hippocampus and cerebral cortex [19, 119–121], but these effects can be blocked in mice with a conditional knockout that reduces levels of BDNF in forebrain regions [122]. Similarly, chronic peripheral subcutaneous administration of BDNF to rats effectuated increased levels and signaling of BDNF along with antidepressant effects, that is, increased mobility in the forced swim test, increased sucrose consumption, decreased latency in the novelty-induced hypophagia test, and increased time spent in the open arms of an elevated plus maze [123]. Rodents overexpressing BDNF or TrkB also exhibit increased resistance and resilience to stress and depressive-related symptoms, that is, decreased immobility in forced swim [124, 125].

The robust effects between stress and BDNF levels are clearly apparent in the hippocampus in the subgranular zone [126]. Neurogenesis in the adult brain is a form of experience-dependent plasticity whereby stem cells within distinct regions (the subgranular zone of the hippocampus and subventricular zone) give rise to new neurons [127]. The 20,000,000 neurons generated over the course of a lifetime have different fates. Some newly born neurons migrate to the granule cell layer, develop a dendritic tree, and send their axon into the mossy fiber pathway [127, 128] to enhance the functional capacity of neural circuitry that is important for learning, memory, and emotional regulation [129–131] in an environmentally dependent manner [132]. Neurons that fail to accomplish this experience a different fate: death. Thus, the proliferation and survival of new neurons in the hippocampus is vitally important for persons with MDD [133], particularly given that the elevations in glucocorticoid levels that cooccur with MDs reduce levels of BDNF and rate of neurogenesis and induce the retraction of dendrites [64].

Fortunately, antidepressant drugs increase levels of BDNF in the hippocampus [19, 21], neurogenesis [134], and hippocampal cell survival rates [135]. The temporal profile of these effects is congruent with the temporal profile of clinical effectiveness of antidepressant drugs, suggesting a similarity between mechanisms [19, 35]. Other preclinical studies suggest that the degree of dendritic branching and the number of spines in hippocampal neurons increase following the restoration of BDNF levels [19, 35, 136–138]. Interestingly, selective deletion of the BDNF gene in the rodent hippocampus attenuates antidepressant efficacy as measured by the elevated plus maze, fear conditioning, sucrose preference, and forced swim tests [139]. Together, these findings suggest that altered expression of BDNF and dysregulation of neurogenesis in the hippocampus may effectuate maladaptive changes in neural networks that are implicated in MDD pathophysiology and, by corollary, that antidepressants may reverse these maladaptive changes.

Subsequent study has revealed that the relationship between BDNF and depressive symptoms is more nuanced in other brain regions. In contrast to the effects seen in the hippocampus, infusion of BDNF to the ventral tegmental/nucleus accumbens area increased depression-like behavior (shorter latency to immobility in the forced swim test) [140] through mechanisms that may involve maladaptive learning. Berton and colleagues demonstrated that chronic stress increased BDNF levels within the nucleus accumbens [141], whereas virally mediated knockdown of BDNF in this region reduced social aversion following chronic social defeat [141], suggesting that increased BDNF in the ventral tegmental area and nucleus accumbens is positively associated with plasticity-induced aversive learning [142–144]. Finally, rodents with a knockdown of the BDNF gene in the ventral tegmental area consumed greater amounts of high-fat diet foods [145], whereas Goto-Kakizaki rats administered intracerebroventricular injections of BDNF exhibited suppressed feeding [146], a finding that underscores the modulating effects of BDNF on feeding behaviors and its interactions with the mesolimbic dopaminergic reward system [147].

Admittedly, central reductions in BDNF are not sufficient to produce depressive-like behaviors in all animals. Rather, reductions of BDNF appear to increase susceptibility to the deleterious effects of stress: exposure of BDNF heterozygous knockout mice to stress induces depressive-like behavior, as does the blockade of BDNF-TrkB signaling following stress [148]. These studies suggest that the anti- or prodepressive effects of BDNF depend upon the brain region affected and offer evidence that selectively targeting BDNF levels in key brain regions may benefit patients affected by MDs. These studies also demonstrate that the effects of BDNF are region- and circuit-specific and cannot be extended arbitrarily to other brain regions.

## 6. BDNF, Plasticity, and Neuroprotection in MDD

It has been proposed that BDNF signaling is a prime mediator of activity-dependent neural plasticity and the resculpting of MD-related circuits [149–151]. Neuroimaging studies have revealed functional deficits in cognitive and affective processing during the early phases of illness [152–155], changes that become increasingly impaired with illness progression [156], and the emergence of structural impairments in the frontal cortex and hippocampus [157–160]. Together, the functional and structural deficits disrupt cognitive and affective regulation that is dependent upon circuit-level integrity of the prefrontal-thalamo-limbic and limbic-striatal-pallidal-thalamic systems [161]. By corollary, circuit level disruption can impede future learning [162]. Cortical regions (e.g., dorsolateral prefrontal cortex [163, 164] and anterior cingulate cortex [165]) comprise the cognitive control network, whereas the subcortical regions (e.g., hippocampus [166], amygdala [167], parahippocampal gyrus [168], caudate nucleus [169], posterior cingulate cortex [168], and thalamus [170]) comprise the affective processing network. Persons who are depressed exhibit impairments in the cognitive control network as evidenced by their inability to disengage from negative stimuli, a task that requires top-down regulation by cortical regions [164, 171]. They may also exhibit impairments in the affective control network as evidenced by hyperactivity of the amygdala [156, 172] and hippocampus [163] to negative stimuli and recall.

The impairments in structure and function in these areas and circuits putatively arise from a myriad of contributing factors, including altered trophic factor level and function, neurotransmitter level and function, stress regulation, peroxisome proliferator-activated receptor C coactivator alpha, neurogenesis, immune function, antioxidant defense, circadian rhythms, epigenetic modifications, and maintenance of telomere length [173]. Within this context, decrements in BDNF are not sufficient to effectuate depression in humans per se. Rather, adequate levels of BDNF effectuate activity-dependent neuronal plasticity that is requisite for the maintenance of basal neuronal and circuit function [174] and for making adaptive responses to endogenous and exogenous stressor challenges [27, 175], particularly during chronic stress [88] and depressive states [87].

Inherent to the depressive state is the inability to return to normal circuit function following the abatement of stressful situations (either psychological or physical in nature), a phenomenon that likely reflects reduced plasticity. Hippocampal atrophy and disconnected brain circuits become increasingly resistant to change in the absence of exogenous interventions that promote recovery. Part of this resistance is the result of the disconnection and loss of function that occurs [19, 24, 176, 177] secondary to synaptic decrements [178, 179].

Synapses typically exhibit plasticity, a state where their function and structure are modified in response to activity and factors in the cellular milieu. LTP is one form of functional synaptic plasticity, wherein connections between synapses become strengthened with activity, a process that is fundamental to learning and memory [180]. Yet, requisite for the strengthening of LTP is the presence of mature BDNF [181]. LTD is another form of functional plasticity, where a set of synapses display a reduced capacity to elicit a response in one another, a process that is vital to forgetting [182]. Requisite for LTD are adequate levels of pro-BDNF [181]. Working in concert, LTP and LTD regulate homeostatic plasticity and the function of neuronal circuits [149, 183] in emotional circuits. This regulation is accomplished by the ability of high-frequency (but not low-frequency) stimulation to induce the secretion of tissue plasminogen activator, a protease that converts extracellular pro-BDNF to mature BDNF [181]. Not surprisingly, the study of hippocampi in persons with MDs has revealed that decreased volumes are positively associated with symptom severity, duration, and treatment outcomes [184–187].

Fortunately, antidepressant drug administration enhances synaptic turnover [188–190], increases synaptic plasticity gene activation [191], and promotes functional connectivity in the hippocampus [192] following stress, processes that are dependent upon TrkB signaling [188]. Also, antidepressant administration increases phosphorylation of TrkB receptors in the rodent hippocampus and cortex within hours [193, 194] and increases the translocation of TrkB receptors to synaptic sites [195]. Via phosphorylation of BDNF and other mechanisms, antidepressant drugs appear to reactivate neuroplasticity.

Maya Vetencourt and colleagues previously reported that chronic administration of fluoxetine, at a dosage that produced serum fluoxetine levels within the therapeutic range in humans, reinstated ocular dominance plasticity in adulthood and promoted visual recovery in amblyopic adult animals. These effects were accompanied by reduced intracortical inhibition and increased BDNF expression in the visual cortex [196]. Similarly, direct infusion of BDNF into the visual cortex recapitulated the effects of fluoxetine [196], suggesting that the antidepressant drug reinstated critical period-like plasticity in the visual cortex [197].

Kobayashi and colleagues demonstrated that chronic treatment of adult mice with fluoxetine greatly reduced expression of calbindin, a marker for mature granule cells in the hippocampus. Additionally, chronic administration of fluoxetine induced active membrane properties that resembled immature granule cells and concomitantly reduced the synaptic facilitation that is characteristic of mature dentate-to-CA3 signal transmission, suggesting that the drug reversed the established state of neuronal maturation in the adult hippocampus [198].

Karpova and colleagues investigated the effects of antidepressants on behavioral experience by using a fear-conditioning and fear-extinction paradigm in rodents. They combined extinction training with chronic fluoxetine and induced an enduring loss of conditioned fear memory in adult animals, an effect that could not be produced without the drug. Strikingly, fluoxetine administration effectuated synaptic plasticity and facilitated the conversion of the fear memory circuitry to an immature state, effects that were mediated by BDNF. The authors concluded that fluoxetine-induced plasticity permits fear erasure by extinction-guided remodeling of the memory circuitry, suggesting that antidepressant drugs may be used to prime plasticity in circuits prior to psychological rehabilitation to facilitate the reorganization and proper function of MDD networks [199].

In another study, Chollet and colleagues administered patients who suffered a stroke add-on fluoxetine to physical therapy. The results of their double-blind, placebo-controlled trial demonstrated that persons who received early prescription of fluoxetine with physical therapy had enhanced motor recovery after 3 months [200].

These findings support the notion that antidepressant drug mechanisms involve the reactivation of neuroplasticity and facilitation of functional reorganization of the neuronal network when accompanied by environmental enrichment [175, 201]. By corollary, they underscore the importance of resolving stressful situations that initially induced functional and structural impairment in mood-related circuits and of deriving biomarkers that facilitate earlier detection and rehabilitation before the illness gains a strong foothold [202]. Also, this evidence highlights a critical unmet need for new antidepressant therapeutics that exert faster onset of action and greater efficacy. Accordingly, a multiplicity of preclinical and clinical investigations has aimed to understand how therapeutics can be used to harness homeostatic mechanisms that regulate neurotrophin release and function to mitigate MDD-related disease, particularly aerobic PA [129, 173, 203].

## 7. Physical Activity, BDNF, and Neuroplasticity

Convergent evidence demonstrates the positive effects of PA in persons with MDD. PA refers to activities that require energy expenditure and involve bodily movements produced by skeletal muscles [204]. Exercise is a subcategory of PA that entails purposeful, planned, and structured endeavors undertaken to improve physical fitness or skill level [204]. Evidence suggests that PA reduces the risk for MDD [205, 206], mitigates symptoms [207], facilitates recovery [208, 209], lowers the incidence of relapse [210, 211], and decreases overall caregiver burden [212]. Undoubtedly, many of the positive effects of PA on brain health and function derive from its ability to optimize central levels of BDNF, particularly in the hippocampus [130].

Preclinical work demonstrates that chronic PA upregulates the expression of BDNF in the hippocampus of rodents for days [116, 213]. Concomitantly, endurance exercise induces elevations of muscle-derived proteins [proliferator-activated receptors (PGC-1*α*) and FNDC5] that regulate BDNF expression in the rodent hippocampus. The ability of PA to modulate changes in BDNF and PGC-1*α* is relevant for stress-induced depression given their interaction with neuroinflammatory and neuroplasticity pathways [214] via alterations in tryptophan degradation [204, 215] and 5-HT_1A_ receptor activation [216].

A bevy of other work underscores the inextricable relationship between PA, BDNF level optimization, and downstream factors. PA optimizes neurotransmitter system level and function (e.g., glutamate, GABA, serotonin, dopamine, and noradrenaline) [173]. In turn, changes in neurotransmission mediate changes in BDNF gene expression in various brain regions (e.g., hippocampus, nucleus accumbens, and amygdala) [217]. Robust preclinical and clinical work demonstrates that PA increases neurogenesis and plasticity via BDNF-dependent mechanisms, particularly when paired with environmental enrichment [173, 218]. Other work demonstrates that PA attenuates the inflammatory process and induces a more resilient stress response [173]. The ability of PA to mitigate HPA dysregulation is especially important for preventing hippocampal atrophy [219, 220] in persons with affective disorders [221] because chronic exposure of hippocampal neurons to elevated glucocorticoid levels induces a retraction of dendrites and reduction of dendritic spines [222].

Emerging preclinical evidence suggests that PA can mitigate the astrocytic dysfunction seen in MDD. Early work in rodents demonstrated that ablation of astrocytes effectuated a reduction of dentate granule cell density and glutamate transporter expression, changes that negate the ability of these cells to effectively remove glutamate excess from the synaptic milieu [223]. Later work demonstrated that chronic stress reduced the number of astrocytic projections in rodents, whereas environmental enrichment increased the number of astrocytic projections [224], a significant finding given that the extent of astrocyte projections is a marker of well-being in these cells. Bolstering the notion of a link between astrocytic function and BDNF is a preclinical work showing that BDNF infusion attenuates hippocampal glial fibrillary acidic level reductions that were a consequence of chronic unpredictable stress [225]. More recent work showed that rodents exposed to long-term PA (5 days per week × 4 weeks) demonstrate increased BDNF synthesis and release in the dentate gyrus along with altered orientation and morphology of astrocytes, effects that are TrkB-signaling dependent [226]. The latter findings suggest that PA-induced changes in astrocytic projection length and density might enhance glutamate clearance from the synapse and mitigate glutamate excitotoxicity in models of MDD, a notion that awaits further study.

Some evidence suggests a synergistic effect of PA and restricted dietary intake on BDNF upregulation. Alomari and colleagues examined the effects of aerobic PA (voluntary wheel running or forced swimming) plus caloric restriction versus dietary restriction alone on BDNF and learning and memory in rodents. Their results demonstrated that the combination of voluntary PA and caloric restriction effectuated greater increases in BDNF levels in the hippocampus, even though improvements in spatial learning and memory occurred in both the combination and dietary restriction groups [227]. Thus, it seems plausible that multidomain treatments such as PA and dietary modification may be particularly beneficial in persons with MDD who exhibit stress-induced decrements in central and peripheral BDNF levels and carry gene-copy-number variants in the BDNF gene.

Carriers of the BDNF Val66Met polymorphism exhibit decreased activity-dependent secretion in comparison to Val/Val carriers, although the level of constitutive secretion of BDNF protein in hippocampal neurons remains the same [83]. Decreased activity-dependent secretion from the neurons of BDNF Met carriers is functionally significant because most BDNF protein is released from the activity-dependent pathway [83, 228]. The fact that PA modulates BDNF levels and symptoms of depression [229, 230] suggests that BDNF gene interactions with PA may influence depressive symptoms [231]. Bolstering this notion is recent work showing that the BDNF polymorphism moderates the association between PA and depressive symptoms. Higher levels of PA were protective against depressive symptoms for girls with the BDNF Met allele, but not for girls with the Val/Val polymorphism [232].

Further elucidating the association between PA and BDNF are clinical investigations of brain structure and function (see Table 1). These studies reaffirm that chronic aerobic exercise increases peripheral levels of BDNF [233–236], blood volume in the dentate gyrus [237], grey matter in the prefrontal and cingulate cortex [235], size of the right and left hippocampus [238], and memory performance [235, 236] in humans. Encouragingly, increases in hippocampal size are correlated with increased spatial memory performance in persons who are healthy and experiencing neurodegenerative changes [238], suggesting that PA might mitigate the cognitive deficits experienced in MDD. Moderate to high training intensity PA appears requisite for maximal PA effects [239, 240]. Clinical studies have demonstrated that acute aerobic PA at 85% of maximal capacity increased plasma BDNF levels, which is important because plasma BDNF levels are linked to alterations in BDNF levels [241, 242], synaptic plasticity, and learning ability [243], whereas blockade of BDNF on TrkB receptors reduced the effects of PA on synaptic plasticity [244].

Parallel studies have investigated the relationship of PA, BDNF, and depressive symptoms. It has been shown that PA increases BDNF in unmedicated patients with MDD [245] and elderly persons with remitted depression [246]. Examining the effects of exercise augmentation (16 kcal/kg/week × 12 weeks) in persons who experienced a partial response to antidepressants, Toups and colleagues found that persons with higher BDNF levels experienced more rapid symptom relief, suggesting that pretreatments with exercise might improve antidepressant efficacy [247]. Schuch and colleagues investigated the effects of add-on PA (16.5 kcal/kg/week of aerobic exercise 3× per week for approximately 3 weeks) with treatment as usual. They found no additional increase of BDNF in the exercise plus medication group relative to the medication-only group, suggesting a plateau effect of antidepressant drugs on BDNF levels in persons with depression [248]. What remains to be determined is whether this plateau effect exists in carriers of the Met allele.

While it is known that PA increases circulating BDNF levels in healthy humans [249] and that BDNF is vitally important for maintaining affective and cognitive circuit function during health and disease, the source of these increases remains unclear. Some have proposed that platelets are the origin of serum BDNF following exercise [250], but BDNF is also increased in plasma samples, a finding that implicates other sources. Krabbe and colleagues demonstrated cerebral output of BDNF in healthy humans at rest [251], suggesting that exercise-induced alterations in plasma BDNF levels reflect altered release of BDNF from the brain [252]. Exploring the latter notion further, Rasmussen and colleagues used arterial-to-internal jugular venous measurement differences to analyze the contribution of the human brain to plasma BDNF levels at rest and during prolonged whole-body exercise. Their results show that the brain is a significant source of BDNF production at rest and during prolonged exercise, contributing approximately three-quarters of the BDNF to venous circulation [253]. Part of the increase in central BDNF may be a consequence of activated platelets in the cerebral circulation [254] or activity-dependent function in other brain structures (e.g., hippocampus and cortex) [253].

These results suggest that PA effectuates central neuroplastic adaptations via optimization of BDNF levels. The ability of PA to enhance BDNF release and function in the synapse, promote dendritic spine integrity, and activate other cellular pathways that contribute to plasticity [19, 24, 255] is vital for homeostatic processes that are necessary for the maintenance, repair, and reorganization of circuits damaged during depression, effects that recapitulate those of antidepressant drugs. While it remains to be determined whether PA can reactivate neuroplasticity, preliminary work by Eadie and colleagues has demonstrated that long-term PA significantly increased total length and complexity of dendrites, increased the spine density on dendrites, and induced a more immature state of dentate granule cells [137].

## 8. Conclusions and Future Directions

The derivation of an effective treatment for MDD represents an unmet goal. Notwithstanding, considerable progress has been made in better understanding the pathobiological features and processes that contribute to the structural, functional, and circuit disruptions that are endemic to MDD. Herein, biomedical evidence demonstrated that stress-induced depressive pathology contributes to altered BDNF level and function in persons with MDD and, thereby, disruptions in neuroplasticity at the regional and circuit level. By corollary, effective therapeutics that mitigate depressive-related symptoms (e.g., antidepressants and physical activity) will optimize BDNF in key brain regions to promote neuronal health and recovery of function in MDD-related circuits. A better ability to deploy therapeutics that optimize BDNF is needed given evidence that intervention in neurodegenerative processes is more likely to achieve disease modification, while ones deployed later demonstrate a significant but more limited effect after the emergence of neuronal degeneration [256].

Clearly, there is an urgent need to identify how PA can best be translated operationally to influence the health and wellness of brain structure and function [173], particularly by optimizing neuroplasticity mechanisms. This challenge will necessarily entail a better understanding of how the optimum mode, intensity, and duration of PA might alter MDD-related symptoms and pathology. Several studies suggest that exercise interventions that combine multiple modalities (e.g., aerobic and strength-training activities) are more effective at enhancing emotional and cognitive health in humans in comparison to those that emphasize aerobic activities alone. Colcombe and Kramer reported that persons who participated in aerobic and strength-training activities exhibited higher gains in cognition in comparison to those who participated in aerobic activities alone [257]. Smith and colleagues reported that interventions that consisted of aerobic and strength-training activities improved attention, processing speed, and working memory to a greater extent than aerobic exercise alone in both healthy individuals and those with neurodegeneration [258], an effect putatively linked to alterations in hippocampal volume [238, 259]. Supporting the latter notion is evidence that decrements in hippocampal size are linked to neurodegenerative progression, whereas the reversal of neurodegenerative progression has been linked to improvements in hippocampal volume [219, 220]. Indeed, aerobic exercise of moderate intensity for 12 months improved memory and hippocampal size in healthy older adults, effectively reversing age-related loss of volume by one to two years [238]. Directly applying the aforementioned, Makizako and colleagues demonstrated that hippocampal volume was directly linked to improved memory in humans and that greater durations of moderate PA could effectuate greater increases in hippocampal volume and memory [259].

Altogether, the data presented here suggests that moderate PA—a target that is practical, well tolerated, and likely to optimize exercise adherence—optimizes BDNF and plasticity, particularly in persons with depression. PA's relative low-risk profile, ease of implementation, and absence of side effects [260] have led to the incorporation of PA into basic clinical management protocols for MDs [261, 262]. Undoubtedly, future efforts to improve population health should consider the ability of lifestyle factors to prevent and treat mental disorders [263] by optimization of neuroplasticity substrates, particularly when coupled with rehabilitation.

## Figures and Tables

**Table 1:** Effects of physical activity on brain-derived neurotrophic factor (BDNF).

Reference	Sample	Treatment	Assessment outcome
[233]	13 young, healthy men	Moderate-intensity aerobic PA 4 d/wk for 5 wks	↑ plasma BDNF
[234]	7 healthy, sedentary males	Aerobic PA 7 d/wk for 12 wks	↑ plasma BDNF
[238]	60 older adults	Aerobic PA 3 d/wk for 60 wks	↑ BDNF and ↑ hippocampal volume
[236]	47 healthy, sedentary males	Aerobic PA 3 d/wk for 5 wks	↑ serum BDNF following PA and ↑ memory on face name matching
[235]	62 healthy, sedentary males	Moderate-intensity aerobic PA for 2 wks	↑ serum BDNF following PA and ↑ memory on face name matching
[247]	104 persons with partial response to antidepressants	Add-on high (16 kcal/kg/week) or low (4 KKW) PA for 12 wks to standard depression care	Persons entering with ↑ BDNF levels exhibited ↑ rate of response to antidepressants
[248]	15 severely depressed adults	Add-on aerobic PA 16 kcal/kg/week for 3 d/wk for 3 wks to standard care for depression or medication-only group	Similar ↑ in BDNF in aerobic PA and medication-only group, but ↓ in oxidative stress markers seen only in PA group

## Abbreviations

BDNF: Brain-derived neurotrophic factor
GABA: *γ*-Aminobutyric acid
LTD: Long-term depression
LTP: Long-term potentiation
MDD: Major depressive disorder
NMDA: N-Methyl-D-aspartate
PA: Physical activity
TrkB receptors: Tropomyosin receptor kinase B.
